# Prognostic value and immune infiltration of novel signatures in colon cancer microenvironment

**DOI:** 10.1186/s12935-021-02342-8

**Published:** 2021-12-18

**Authors:** Yilin Lin, Xiaoxian Pan, Zhihua Chen, Suyong Lin, Zhanlong Shen, Shaoqin Chen

**Affiliations:** 1 Department of Gastroenterological Surgery, The First Affiliated Hospital of Fujian Medical University, No. 20, Chazhong Road, Taijiang, Fuzhou, Fujian China; 2 Department of Gastroenterological Surgery, Peking University People’s Hospital, 11 Xizhimen South Street, Xicheng, Beijing, China; 3 Department of Radiotherapy, The First Affiliated Hospital of Fujian Medical University, Fuzhou, Fujian China

**Keywords:** Colon cancer, Tumor microenvironment, Noncoding RNA, H19, Immune infiltration, Survival

## Abstract

**Background:**

Growing evidence has shown that the prognosis for colon cancer depends on changes in microenvironment. The purpose of this study was to elucidate the prognostic value of long noncoding RNAs (lncRNAs) related to immune microenvironment (IM) in colon cancer.

**Methods:**

Single sample gene set enrichment analysis (ssGSEA) was used to identify the subtypes of colon cancer based on the immune genomes of 29 immune signatures. Cox regression analysis identified a lncRNA signatures associated with immune infiltration. The Tumor Immune Estimation Resource database was used to analyze immune cell content.

**Results:**

Colon cancer samples were divided into three subtypes by unsupervised cluster analysis. Cox regression analysis identified an immune infiltration-related 5-lncRNA signature. This signature combined with clinical factors can effectively improve the predictive ability for the overall survival (OS) of colon cancer. At the same time, we found that the expression of H19 affects the content of B cells and macrophages in the microenvironment of colon cancer and affects the prognosis of colon cancer. Finally, we constructed the H19 regulatory network and further analyzed the possible mechanisms. We found that knocking down the expression of H19 can significantly inhibit the expression of CCND1 and VEGFA. At the same time, the immunohistochemical assay found that the expression of CCND1 and VEGFA protein was significantly positively correlated with the infiltration of M2 type macrophages.

**Conclusion:**

The findings may help to formulate clinical strategies and understand the underlying mechanisms of H19 regulation. H19 may be a biomarker for targeted treatment of colon cancer.

**Supplementary Information:**

The online version contains supplementary material available at 10.1186/s12935-021-02342-8.

## Background

Colon cancer has become the most common gastrointestinal tumor. According to research reports, the incidence of colon cancer worldwide is 10.2%, ranking third; the death rate of colon cancer is 9.2%, ranking second [1]. Although there have been some breakthroughs in the research on the mechanism of colon cancer occurrence and development [2–4], the carcinogenic factors are still unknown. At present, most of the clinical prognosis of colon cancer is based on the tumor-node-metastasis (TNM) stage [5], and numerous studies have found that new assessment methods may be more suitable for cancer prognosis analysis than a single TNM stage [6–8]. Considering the high morbidity and mortality of colon cancer, finding a new method that can improve the prognostic value of colon cancer is very urgent. A new method could help inform efficient decisions and treatment options, and it can also provide new insights into colon cancer development mechanisms.

The tumor microenvironment (TME) is composed of fluid, immune cells, stromal cells, extracellular matrix, and numerous cytokines and chemokines [9]. The levels of these cells and molecules reflect the evolutionary nature of cancer and promote tumor immune escape, tumor growth and metastasis [10]. Components of the TME can divide cancer into more specific subtypes, which may better assess the prognosis and treatment of cancer [11, 12]. Understanding the composition and function of TME molecules is essential for the effective management of cancer progression and the immune response [13].

With the development of technology, transcriptomics sequencing has been widely used in disease research [14–16]. Transcriptomic analysis has greatly improved our understanding of disease occurrence and development [17, 18]. LncRNA is a non-coding RNA that plays a key role in the development of diseases [19, 20]. The application of sequencing technology has led to an increasing number of cancer-related lncRNAs. A study has reported that noncoding RNAs are potential mediators of anticancer immunotherapy [21]. Xu et al. found that lncRNA SATB2-AS1 affects colon cancer immune microenvironment and inhibits colon cancer progression [22]. Related studies have reported lncRNA markers as prognostic targets for cancer [23, 24]. A study demonstrated that downregulation of lncRNA HOTAIRM1 promotes monocyte/dendritic cell differentiation [25]. NKILA lncRNA promotes tumor immune escape by sensitizing T cells to activation-induced cell death [26]. Increased expression of lncRNA GAS5 may enhance the killing effect of NK cells on liver cancer [27]. Lnc-C/EBPβ was shown to negatively regulate the inhibitory function of myeloid-derived suppressor cells [28]. Fitzgerald et al. showed that lncRNAs play an important role in the development and activation of immune cells [29]. These studies indicate that lncRNAs play an important role in the regulation of the components of the tumor microenvironment. Imbalance in the expression of these lncRNAs may be an important mechanism for tumor immune escape.

In this study, we used ssGSEA to assess the enrichment level of immune signatures in colon cancer [30, 31]. Colon cancer samples were divided into three subtypes by unsupervised cluster analysis. Cox regression analysis identified an immune infiltration-related 5-lncRNA signature. This signature combined with clinical factors can effectively improve the predictive ability for the OS of colon cancer. At the same time, we found that the expression of H19 affects the content of B cells and macrophages in the microenvironment of colon cancer and affects the prognosis of colon cancer. This study may help develop clinical strategies and provide evidence for finding molecular markers for targeted therapy for colon cancer.

## Materials and methods

### Data acquisition

RNA sequencing data and clinical data of colon adenocarcinoma samples were downloaded from TCGA (https://cancergenome.nih.gov/), based on the Illumina HiSeq 2000 RNA Sequencing platform. The work flow type is counts. This dataset contains 488 colon adenocarcinoma tissues and 42 adjacent normal tissues. Remove incomplete samples of survival data, and finally obtain 447 samples with complete clinical information for subsequent analysis. Ensemble IDs of lncRNA in TCGA database were extracted from the GENCODE project (http://www.gencodegenes.org). R package "limma" and “voom” function are used to normalize the data. We adopted the datasets (GSE17536) from the GEO database. The GSE17536 data were based on GPL570 platforms (Affymetrix Human Genome U133 Plus 2.0 Array, 176 colon patients). Immune-related genes (IRGs) were obtained from the Molecular Signatures Database v4.0 (Immune system process M13664, Immune response M19817; https://www.broadinstitute.org/gsea/msigdb/index.jsp) [32].

### Clustering and evaluation of IM in colon cancer

The enrichment level of 29 immune signatures in each sample was evaluated by ssGSEA. After that, a hierarchical clustering was performed on the enrichment level. ESTIMATE [33] was used to assess the immune score, tumor purity, and stromal score of each sample. The ESTIMATE score is obtained from the stromal score plus the immune score.

### Identification of immune infiltration-related lncRNAs

The samples are hierarchically clustered based on the IM score. Identify the differentially expressed lncRNA in each subtype. A *p* value < 0.05 and |fold change|> 1 were set as the cutoff values. Immune infiltration-related lncRNAs were obtained through the co-expression level of IRGs and lncRNA. |Pearson correlation coefficient|> 0.4 and *p* value < 0.001 were set as the cutoff.

### Construction of an immune infiltration-related lncRNA signature

Univariate Cox analysis was used to screen prognosis-related lncRNAs (*p* value < 0.05 was set as the cutoff). The Akaike information criterion (AIC) had a minimum value of 755.44 as the best cutoff point in multivariate Cox regression analysis. The immune infiltration-related lncRNA signature is expressed as follows: risk score = (coefficient _lncRNA1_ × lncRNA1 expression) + (coefficient _lncRNA2_ × expression of lncRNA2) + … + (coefficient _lncRNAn_ × expression lncRNAn). The samples were divided into high-risk group and low-risk group based on the median risk score. Kaplan–Meier curves were used to assess overall survival. The time-dependent receiver operating characteristic (ROC) curve is used to evaluate the prognostic value of this signature.

Complete information on the 447 patients included relevant clinical data for Cox regression analysis.

### Building a predictive nomogram

Each factor model, clinical model (age, gender and stage) and combination model (age, gender, stage and risk score) were compared with a ROC curve.. The calibration plot was used to investigate the calibration of the nomogram.

### Tumor immune estimation resource (TIMER) database analysis

The TIMER database uses RNA-Seq expression profiling data to detect immune cell infiltration in tumor tissue. TIMER provides the infiltration of 6 types of immune cells (B cells, CD4 + T cells, CD8 + T cells, Neutrphils, Macrophages and Dendritic cells) [34]. This database also contains the expression of gene expression levels in 32 cancers. It also provides the prognostic value of immune cell content in cancer.

### Construction of the lncRNA ceRNA network

The miRcode database was used to predict possible lncRNA binding to bound miRNAs. Upregulated mRNA and downregulated miRNA were screened by comparing normal samples and colon cancer samples based on the TCGA database. The miRDB, TargetScan and miRTarBase databases were used to predict genes that miRNAs might bind. The lncRNA ceRNA network was visualized using Cytoscape 3.7.1.

### Gene set enrichment analysis (GSEA)

To reveal the potential underlying the gene ontology (GO) and Kyoto Encyclopedia of Genes and Genomes (KEGG) pathways of the immune infiltration-related lncRNA signature, GSEA was used to analyze the enrichment terms.

### Drug sensitivity analysis

The GSCALite database (http://bioinfo.life.hust.edu.cn/web/GSCALite/) was used to explore the relationship between molecular expression and drug resistance in tumor cells. Drug resistance analysis of genes was performed based on GDSC/CTRP IC50 drug data. Spearman correlation indicates that gene expression is drug-related. Positive correlation means high gene expression is resistant to drugs.

### Cell culture

Colorectal cancer cell lines RKO, HCT116 and HCT8 were purchased from the Chinese Academy of Sciences Cell Bank. The cells are cultured in DMEM solution containing 10% fetal bovine serum. All cells are cultured at 37˚C containing 5% CO_2_.

### Real-time quantitative polymerase chain reaction (RT-PCR) assay

A total of three si-H19s were purchased from GenePharma (Shanghai, China). Colorectal cancer cells grown (RKO, HCT116 and HCT8) in a six-well plate, and when the cell proliferation density reaches 80%, perform RNA (si-H19) transfection according to the reagent manufacturer's instructions. When 48 h after transfection, RNA was extracted by trizol reagent (ER501-01, TransGen Biotech, Beijing, China). The reverse transcription kit (AE341-02) and amplification kit (AQ101-01) were purchased from TransGen Biotech (Beijing, China). The methods and steps of RNA reverse transcription and amplification were carried out in accordance with the instructions of the reagent manufacturer. GAPDH was used as an internal reference gene. The primer sequences of all genes were shown in Table1.

**Table1:** Primer sequences for all genes

Gene	Primer sequence	
H19	Forward	5′-ATGACATGGTCCGGTGTGAC-3′
	Reverse	5′-GAAACAGACCCGCTTCTTGC-3′
CCND1	Forward	5′-CAGATCATCCGCAAACACGC-3′
	Reverse	5′-AAGTTGTTGGGGCTCCTCAG-3′
VEGFA	Forward	5′-CTGACGGACAGACAGACAGACAC-3′
	Reverse	5′-CGAAGCGAGAACAGCCCAGAA-3′
GAPDH	Forward	5′-AAATCAAGTGGGGCGATGCT-3′
	Reverse	5′-CAAATGAGCCCCAGCCTTCT-3′

### Western blot assay

When si-H19 was transfected for 48 h, add RIPA lysis buffer and protease inhibitor (Solarbio, Beijing, China) to extract total protein. BCA protein quantification kit was used to detect protein concentration (Solarbio, Beijing, China). Add SDS loading buffer to the extracted total protein, and then boil it in 100° water for five minutes for subsequent experiments. The obtained protein was electrophoresed in 12% sodium dodecyl sulfate–polyacrylamide gel electrophoresis (SDS-PAGE). PVDF membrane was used for electroporation. The protein electrophoresis was performed at a stable voltage of 100 V, and the electroporation was performed at a stable current of 300 mA for 90 min. After electroporation, soak the PVDF membrane in 5% skimmed milk and place it on a shaker for half an hour. Then add 5 ml of VEGFA (Abcam, ab52917, 1:1000), CCND1 (Abcam, ab134175, 1:1000) or GAPDH (CST, 5174S, 1:1000) rabbit-derived primary antibody to the PVDF membrane and incubate overnight at 4°. After incubating overnight, collect the primary antibody, add Tris-Buffered Sal ine Tween 20 (TBST) and wash three times for 15 min each time. Add goat anti-rabbit (Solarbio, Beijing, China, 1:5000) and incubate for 1 h, then continue to wash with TBST three times. Finally, add electrochemiluminescence (Solarbio, Beijing, China) liquid to expose in the exposure instrument.

### Immunohistochemical staining assay

The tissue block was embedded in paraffin and continuously cut into 3 µm sections and placed on a glass slide. The slices were baked at 72 °C for 1 h, dewaxed with xylene, and dehydrated with gradient alcohol. After rinsing 5 times with phosphate buffered saline (PBS), 2 min each time (1‰ Tween 20 is added to PBS), then after high-pressure repair, rinse again with PBS 5 times. The processed sections were immersed in a 3% hydrogen peroxide solution, incubated at room temperature for 10 min, and washed with distilled water and PBS. Next, VEGFA, CCND1 or CD163 rabbit-derived primary antibody (Abcam, ab182422, USA; 1:100) were added to the slices in equal proportions, incubated at 37 °C for 1 h, and rinsed with PBS 3 times for 5 min each time. Then incubated with a fluorescein isothiocyanate-conjugated goat anti- rabbit IgG secondary antibody (ZSGB-BIO, Beijing, China) for 30 min. After that, the specific observation and evaluation methods are described in detail in the previously published articles [35].

## Results

### Immunogenomic profiling identifies three colon cancer subtypes

**Fig. 1:**
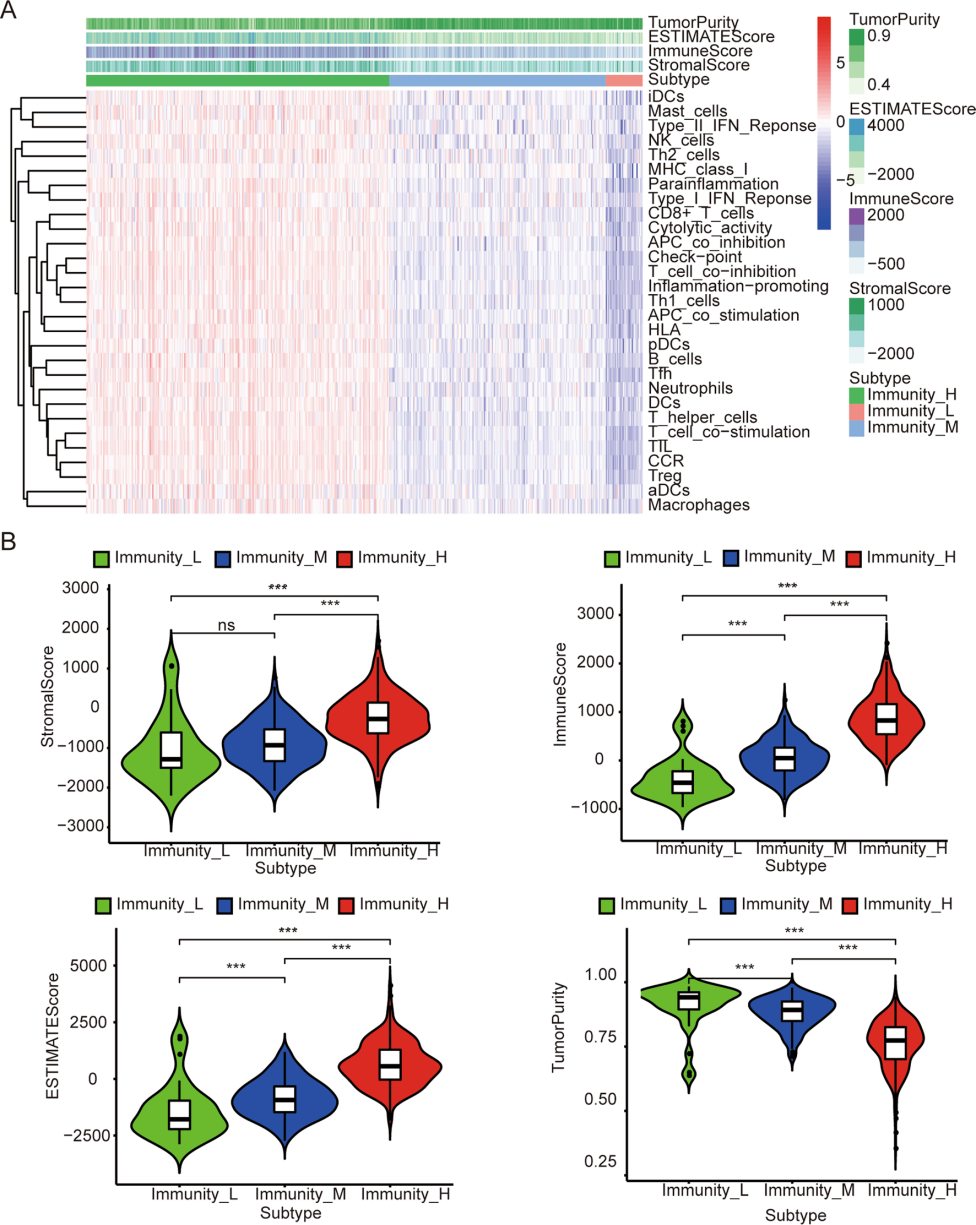
Identification of subtypes of colon cancer based on IM score. **A** The immune cell infiltration level in each subtype, tumor purity, ESTIMATE score, stromal score and immune score were evaluated by ESTIMATE. **B** Comparison of stromal score, immune score, ESTIMATE score and immune score and tumor purity between colon cancer subtypes (Mann–Whitney U test). *P < 0.05, **P < 0.01, ***P < 0.001

**Fig. 2:**
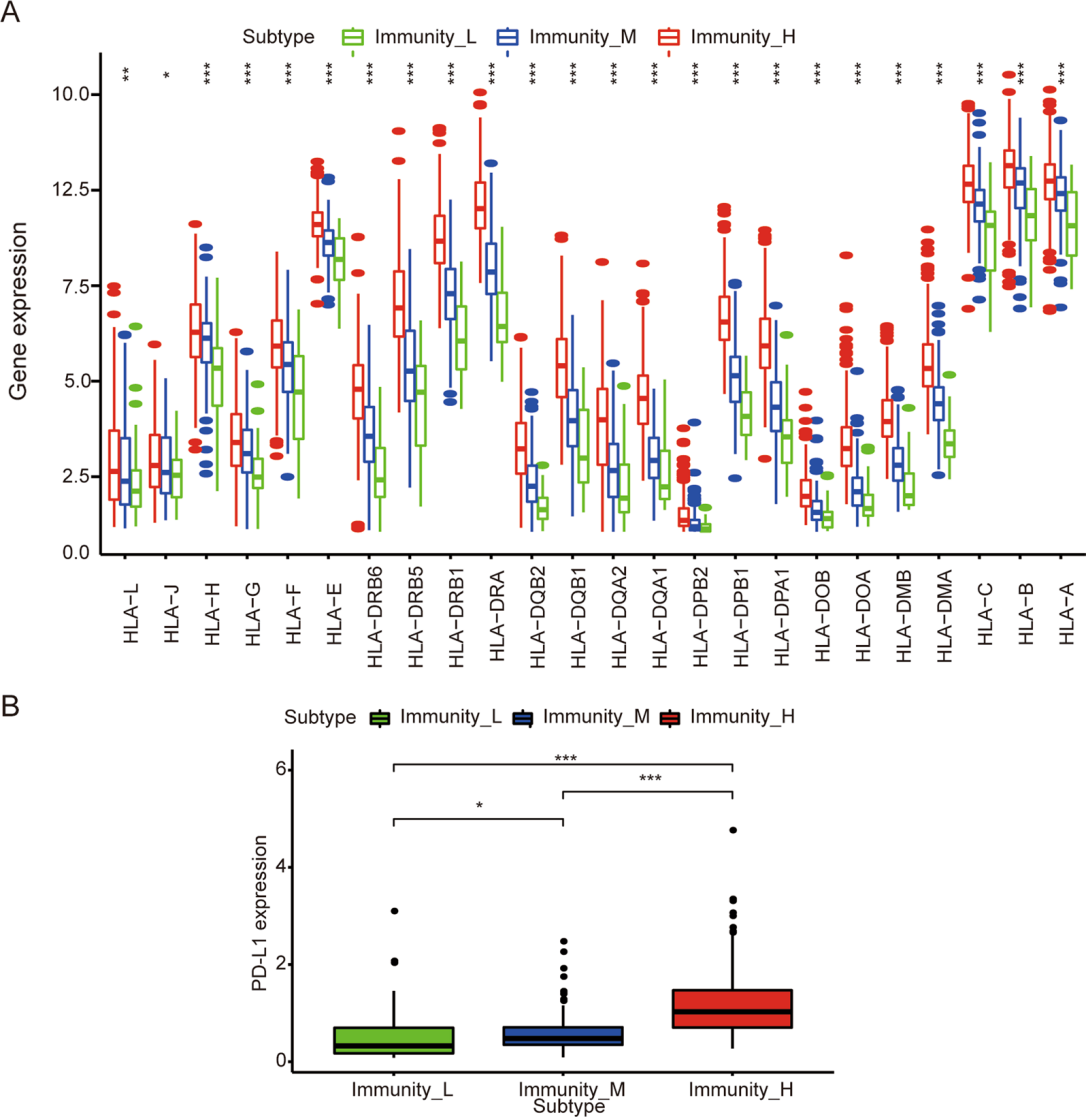
Comparison of HLA gene and PD-L1 expression between colon cancer subtypes. **A** Comparison of the expression levels of HLA genes between colon cancer subtypes (ANOVA test). **B** Comparison of PD-L1 expression levels between colon cancer subtypes (ANOVA test). *P < 0.05, **P < 0.01, ***P < 0.001

The score for each sample by ssGSEA is based on 29 immune signatures (Additional file 1: Table S1). According to the cluster analysis of the scores of the samples, we define three subtypes as high immunity (Immunity_H), medium immunity (Immunity_M) and low immunity (Immunity_L). A heatmap shows the infiltration levels and scores of each sample of immune cells in the three subtypes (Fig. 1A). We found that the stromal score, immune score and estimated score in the Immunity_H group were significantly higher than those of Immunity_L and Immunity_L (P < 0.001) (Fig. 1B). The tumor purity score has the opposite trend. Moreover, most HLA genes are significantly overexpressed in Immunity_H (Fig. 2A). In addition, PD-L1 gene was significantly overexpressed in Immunity_H (Fig. 2B). This indicates that Immunity_H may benefit more from immune checkpoint inhibitor therapy.

### Identification of an immune infiltration-related lncRNA signature

**Fig. 3:**
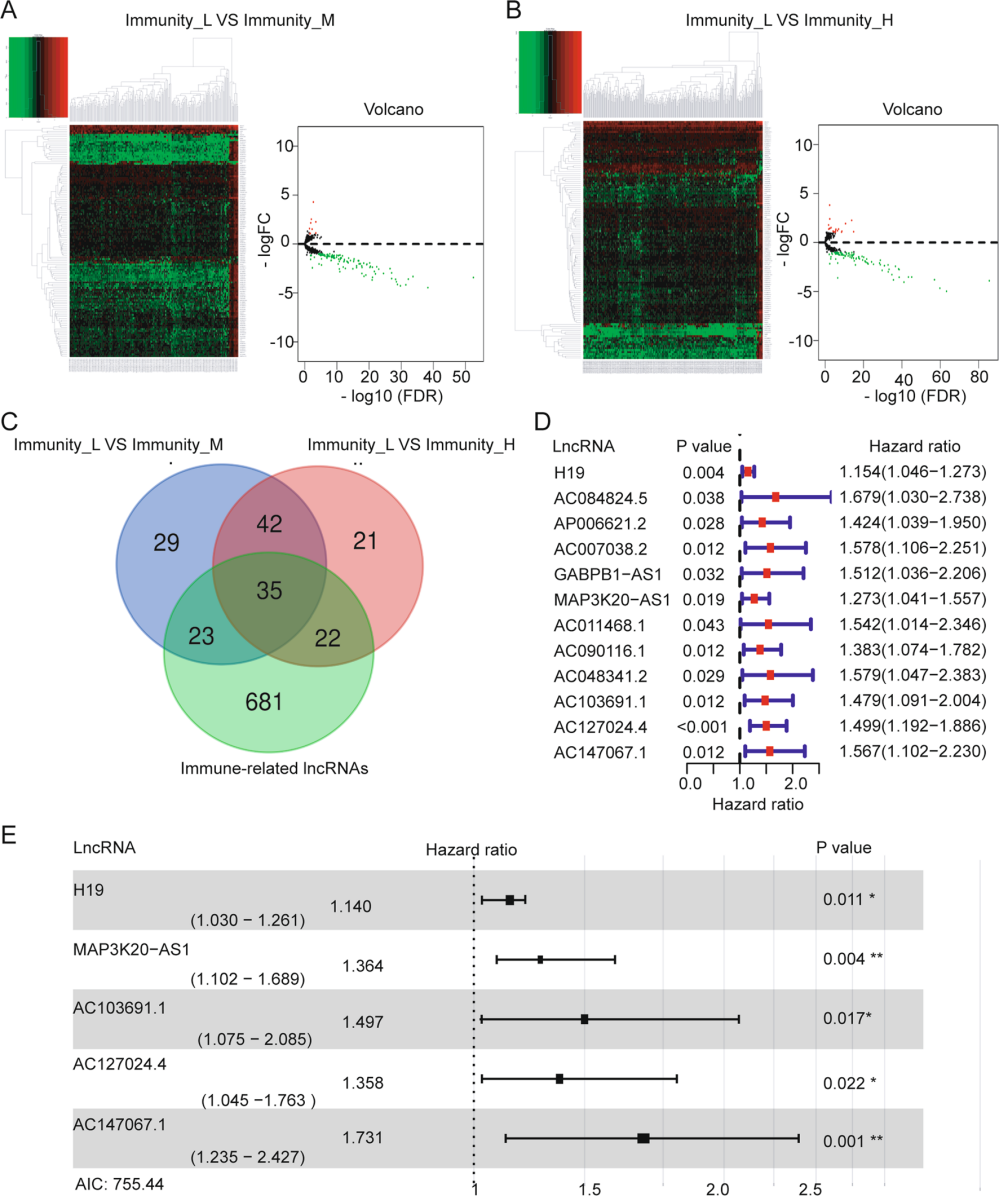
Identification and construction of an immune infiltration-related lncRNA signature. **A** Differentially expressed lncRNAs between Immunity_L and Immunity_M. **B** Differentially expressed lncRNAs between Immunity_L and Immunity_H. The expression of each differentially expressed lncRNA is shown in the heatmap. Volcano plots indicate the fold change of each differentially expressed lncRNA. Red dots indicate highly expressed lncRNAs, and green dots indicate poorly expressed lncRNAs. **C** Intersecting lncRNAs in three sets. A total of 35 differentially expressed lncRNAs were screened. **D**, **E** Through univariate Cox regression and multivariate Cox regression analysis, five immune infiltration-related lncRNAs were used to evaluate colon cancer prognosis

One hundred twenty-nine differentially expressed lncRNAs were screened by comparing Immunity_L and Immunity_M, with low expression of 120 lncRNAs and high expression of 9 lncRNAs (Fig. 3A) (Additional file 2: Table S2). One hundred and twenty differentially expressed lncRNAs were screened by comparing Immunity_L and Immunity_H, with low expression of 102 lncRNAs and high expression of 18 lncRNAs (Fig. 3B) (Additional file 3: Table S3). Seven hundred sixty-one immune-related lncRNAs were screened for coexpression analysis with immune-related genes (Additional file 4: Table S4). From the Venn diagram, we obtained 35 lncRNAs for subsequent analysis (Fig. 3C).

**Fig. 4:**
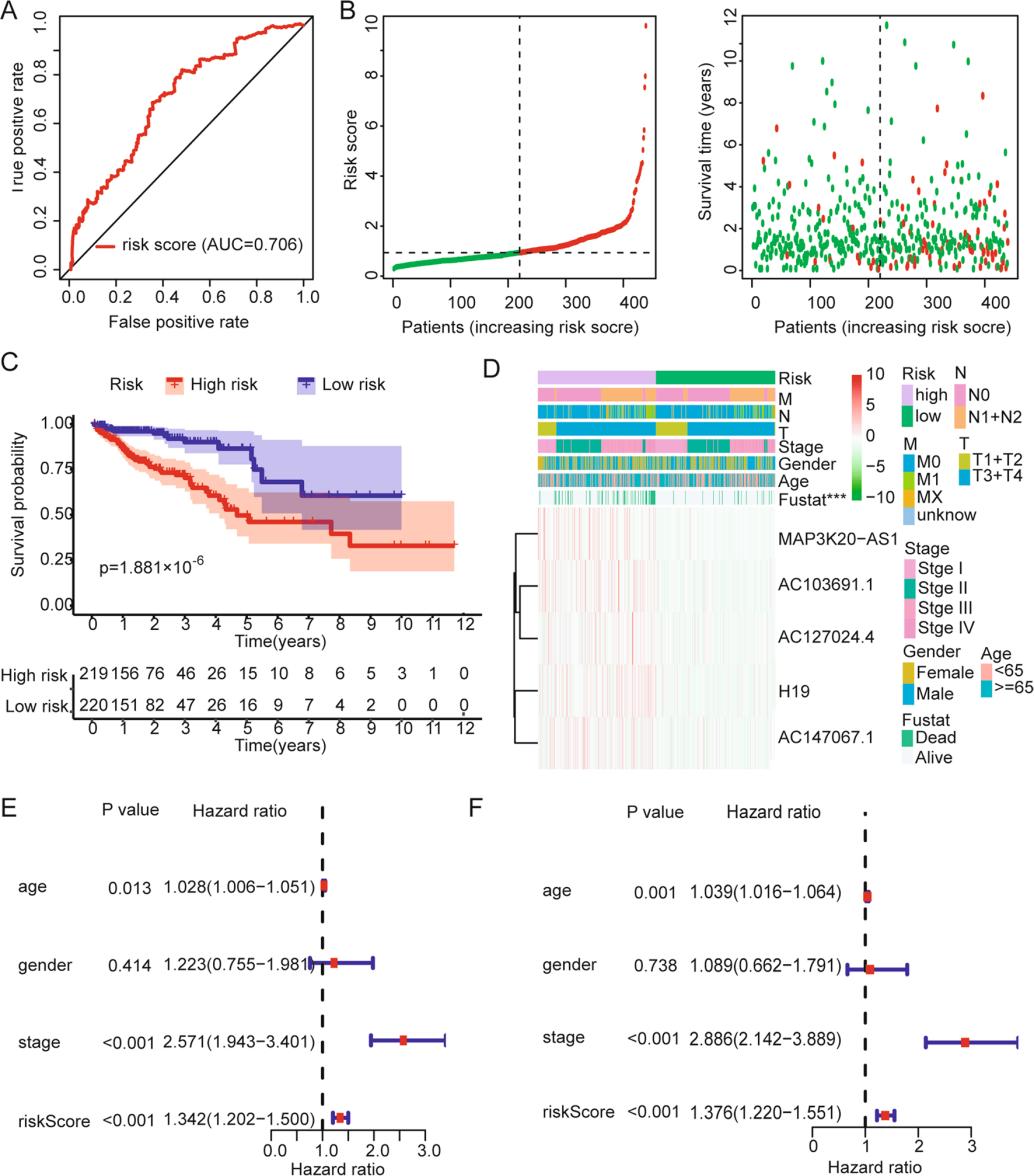
Time-dependent ROC analysis, risk score analysis, Kaplan–Meier analysis, and univariate and multivariate Cox regression analysis for the signature in colon cancer. **A** ROC (3-year) analysis of the signature. **B**, **C** Risk score and Kaplan–Meier analysis for patients in high-risk and low-risk groups by signature. **D** A heatmap of lncRNA expression and clinical features for patients in high-risk and low-risk groups. **E**, **F** Univariate and multivariate Cox regression analysis to identify independent prognostic factors for colon cancer

Thirty-five differentially expressed lncRNAs were analyzed by univariate Cox regression analysis. We identified 12 immune-related lncRNAs associated with OS (Fig. 3D). The results show that the hazard ratios (HRs) of these 12 lncRNAs are all greater than 1, indicating that their high expression may be related to poor prognosis of colon cancer. Next, we analyzed these 12 lncRNAs by multivariate regression. With an AIC value of 755.44 as the best cutoff point, we finally identified 5 lncRNAs that were used to evaluate the OS of colon cancer patients (Fig. 3E, Table 2). The 5 immune-related lncRNAs included H19, MAP3K20-AS1, AC103691.1, AC127024.4, and AC147067.1. Risk score = 0.131 × expression of H19 + 0.311 × expression of MAP3K20-AS1 + 0.403 × expression of AC103691.1 + 0.306 × expression of AC127024.4 + 0.549 × expression of AC147067.1. Patients were divided into high-risk and a low-risk groups with an optimal cutoff of 0.938 for risk score. The area under the ROC curve (AUC) for 3-year OS was 0.706 (Fig. 4A). These results indicate that the prognostic model is moderately sensitive and specific. As the risk score increased, the patient mortality rate increased gradually (Fig. 4B). The OS was significantly poorer in the high-risk group than that in the low-risk group (p < 0.0001; Fig. 4C). The heatmap shows that five lncRNAs are significantly highly expressed in the high-risk group, but there are no significant differences in clinical characteristics between the high-risk group and the low-risk group (Fig. 4D). Univariate Cox regression (Fig. 4E) and multivariate Cox regression (Fig. 4F) analysis showed that the p-values of age, stage, and risk scores were all less than 0.05, and the HR values were all greater than 1. These three factors may be independent prognostic factors of colon cancer, and their increased expression values may lead to poor OS for colon cancer patients.

**Table 2:** Univariate and multivariate Cox regression analysis of immune infiltration-related lncRNAs in colon cancer

Variables	Univariate analysis		Multivariate analysis		
	HR 95% CI	P value	Coefficient	HR (95% CI)	P value
H19	1.154 (1.046–1.273)	0.004	0.131	1.140 (1.030–1.261)	0.011
MAP3K20-AS1	1.273 (1.041–1.557)	0.019	0.311	1.364 (1.102–1.689)	0.004
AC103691.1	1.479 (1.091–2.004)	0.012	0.403	1.497 (1.075–2.085)	0.017
AC127024.4	1.499 (1.192–1.886)	< 0.001	0.306	1.358 (1.045–1.763)	0.022
AC147067.1	1.567 (1.102–2.230)	0.012	0.549	1.731 (1.235–2.427)	0.001

### Constructing a predictive nomogram in colon cancer

**Fig. 5:**
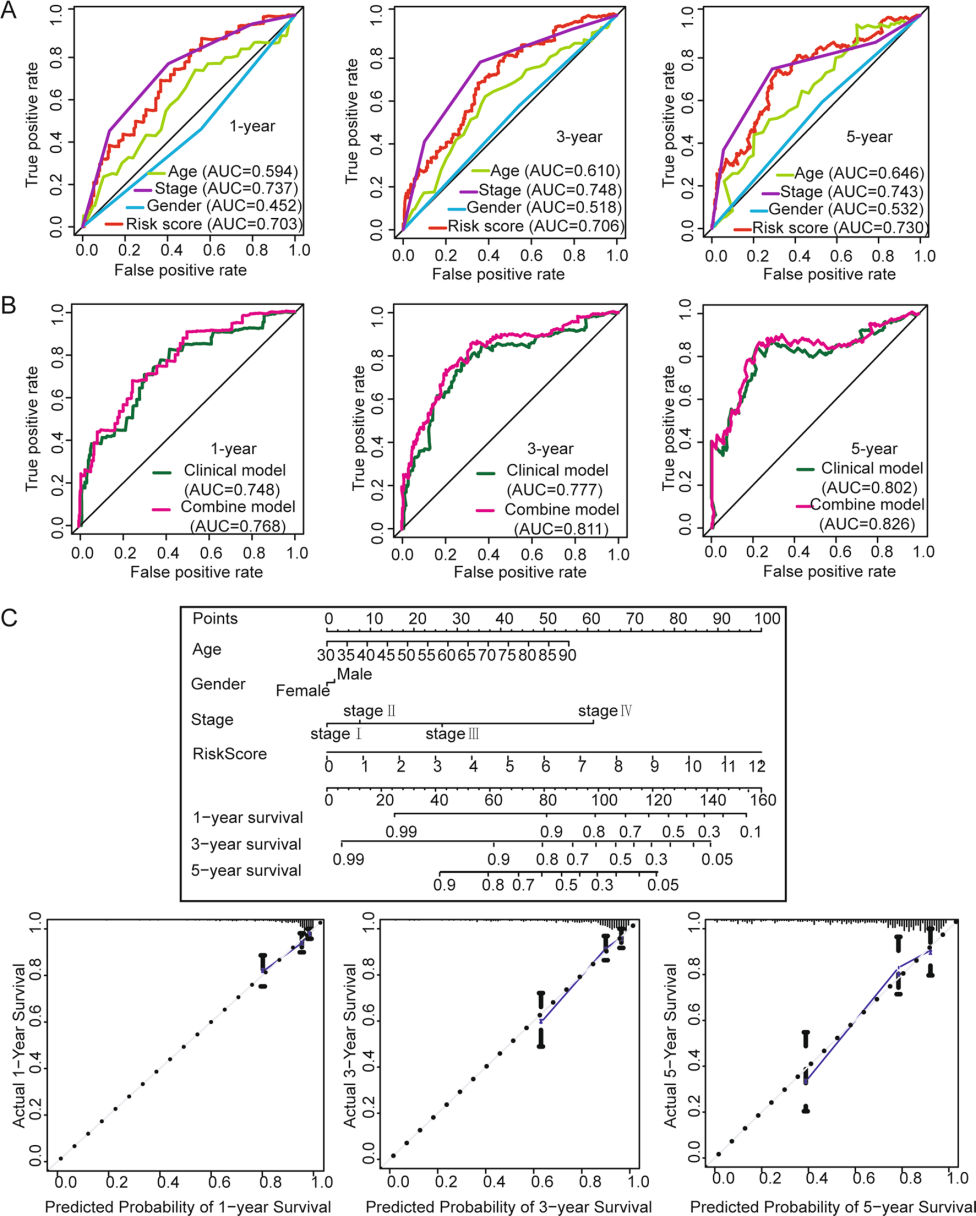
Building of the nomogram predicting overall survival for colon cancer patients. **A** The ROC curves of each factor compared for 1-year, 3-year, and 5-year OS in colon cancer. **B** The time-dependent ROC curves of the clinical model and combined model compared for 1-year, 3-year, and 5-year overall survival in colon cancer. **C** The nomogram plot was built based on all factors in the combined model. The calibration plot for validation of the nomogram

To construct a predictive nomogram, we plotted the ROC curves to assess each factor for predicting the OS of colon cancer at 1 year, 3 years and 5 years (Fig. 5A). The AUCs for 1-year, 3-year and 5-year OS were 0.594, 0.610, and 0.646, respectively, in the age model. The AUCs for 1-year, 3-year and 5-year OS were 0.737, 0.748, and 0.743, respectively, in the stage model. The AUCs for 1-year, 3-year and 5-year OS were 0.452, 0.518, and 0.532, respectively, in the gender model. The AUCs for 1-year, 3-year and 5-year OS were 0.703, 0.706, and 0.730, respectively, in the risk score model (an immune infiltration-related lncRNA signature). This result shows that the stage model and risk score model may have moderately accurate prediction capabilities, followed by the age model and the gender model. In the preliminary assessment of prognosis, age, gender, and stage factors were used to initially assess the prognosis of patients with colon cancer, so we constructed a clinical model including age, gender and stage. A combined model including age, gender, stage and risk score was also constructed (Fig. 5B). The AUCs for 1-year, 3-year and 5-year OS were 0.748, 0.777, and 0.802, respectively, in the clinical model. The AUCs for 1-year, 3-year and 5-year OS were 0.768, 0.811, and 0.826, respectively, in the combined model. This result shows that the combined model has more accurate predictions than the other models. Finally, a nomogram was built using the combined model (Fig. 5C). Calibration plots showed that the performance of the nomogram was best in predicting 1-year, 3-year and 5-year OS.

In summary, our predictive model may increase the predictive sensitivity and specificity of traditional clinical models and provide some benefits that may help clinical management.

### GSEA and the relationship between lncRNAs and clinical characteristics

**Fig. 6:**
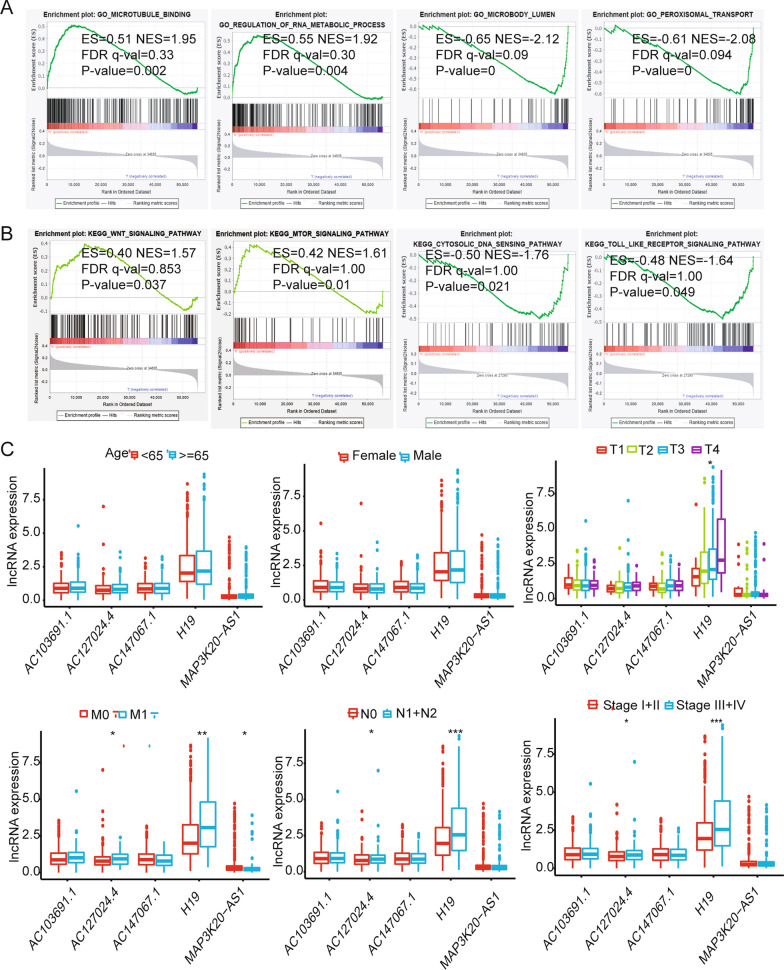
GSEA and the relationship between lncRNAs and clinical characteristics. **A** The main GO enrichment items in the high-risk patient group were microtubule binding and regulation of RNA metabolic process. The main GO enrichment items in the low-risk patient group were microbody lumen and peroxisomal transport. **B** The main enrichment items of KEGG in high-risk patients are the WNT signaling pathway and mTOR signaling pathway. The main enrichment items of KEGG in low-risk patients are the cytosolic DNA sensing pathway and Toll-like receptor signaling pathway. **C** Comparison of each lncRNA expression level between clinical characteristics (age, gender, T stage, M stage, N stage and clinical stage (ANOVA test)). *P < 0.05, **P < 0.01, ***P < 0.001

The GSEA results show that the main GO enrichment items in the high-risk patient group were microtubule binding and regulation of RNA metabolic process (Fig. 6A). The main GO enrichment items in the low-risk patient group were microbody lumen and peroxisomal transport (Fig. 6A). The main enrichment items of KEGG in high-risk patients were the WNT signaling pathway and mTOR signaling pathway (Fig. 6B). The main enrichment items of KEGG in low-risk patients were the cytosolic DNA sensing pathway and Toll-like receptor signaling pathway (Fig. 6B). When comparing each lncRNA expression level between clinical characteristics, we found that the expression of these five lncRNAs was not significantly different by age or gender. The expression of AC127024.4 is related to the distant metastasis (M), lymphatic node metastasis (N) stage and clinical stage of colon cancer (*p* < 0.05) (Fig. 6C). The expression of MAP3K20-AS1 is related to the distant metastasis of colon cancer (*p* < 0.05) (Fig. 6C). However, the expression of H19 was significantly related to the tumor invasion depth of colon cancer (T, *p* < 0.05), distant metastasis (M, *p* < 0.01), lymphatic node metastasis (N, *p* < 0.001) and TNM stage (*p* < 0.001) (Fig. 6C). This indicates that H19 expression may be related to the development and prognosis of colon cancer.

### Relationship between H19 expression and immune cell infiltration level

**Fig. 7:**
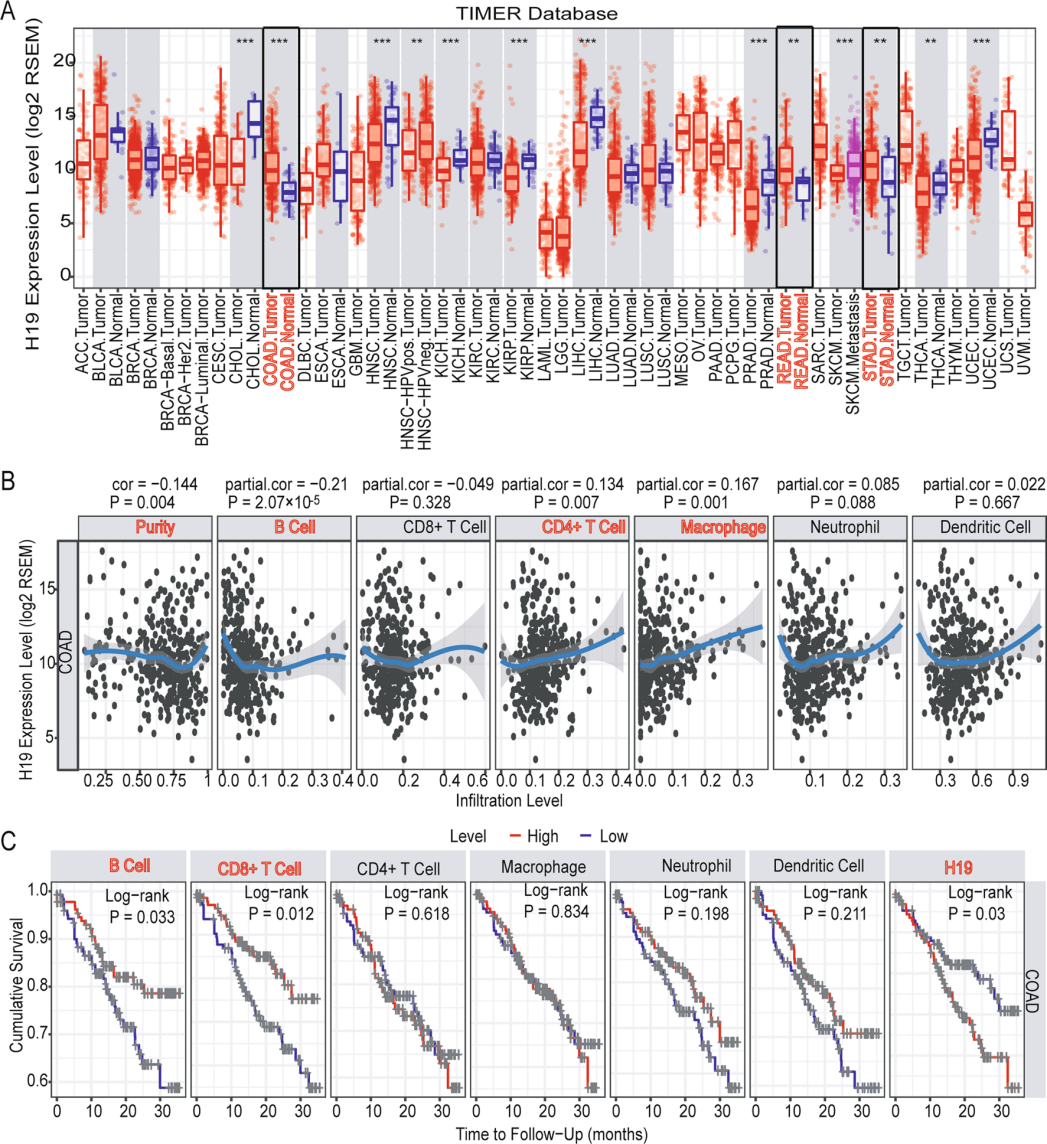
H19 expression and immune cell infiltration levels based on the TIMER database. **A** H19 expression levels in 32 cancers. The results show that H19 is significantly overexpressed in gastrointestinal tumors. **B** Correlation analysis between H19 expression and six immune cell infiltration levels in colon cancer. **C** Prognostic analysis of H19 expression and six immune cell infiltration levels in colon cancer. *P < 0.05, **P < 0.01, ***P < 0.001

To further study the role of H19 in colon cancer, we analyzed the expression levels of H19 in 32 cancers using the TIMER database (Fig. 7A). The results showed significantly higher expression in colon cancer (*p* < 0.01), gastric cancer (*p* < 0.01), and rectal cancer (*p* < 0.01). H19 may play a role as a cancer-promoting factor in gastrointestinal tumors. Next, we analyzed the correlation between the expression of H19 and the invasion of immune cells using the TIMER database (Fig. 7B). The results showed that the expression of H19 was negatively correlated with tumor purity and B-cell invasion, which was statistically significant (*p* < 0.05). The expression of H19 was positively correlated with the infiltration of CD4 + T cells and macrophages, which was statistically significant (*p* < 0.05). Survival prognosis analysis showed that high expression of H19 had poor OS (Fig. 7C, p < 0.05), but high B-cell infiltration and high CD8 + T cell infiltration had good OS (Fig. 7C, p < 0.05).

**Fig. 8:**
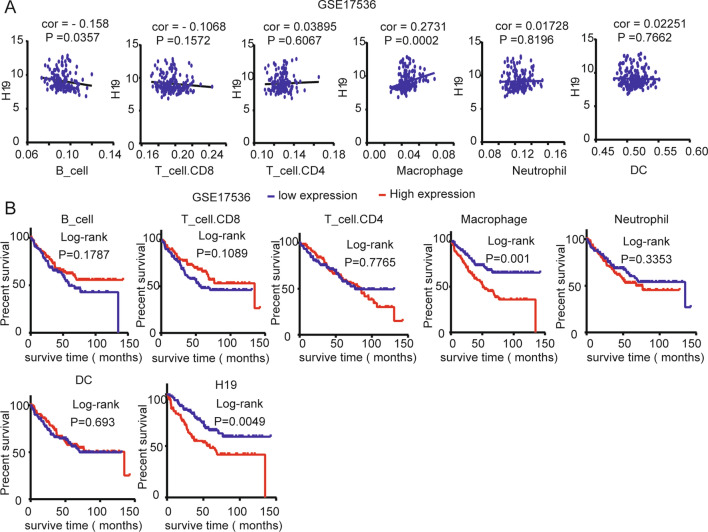
Validation of H19 expression and immune cell infiltration levels based on the GSE17563 data set. **A** Correlation analysis between H19 expression and six immune cell infiltration levels in the GSE17563 dataset. **B** Prognostic analysis of H19 expression and six immune cell infiltration levels in the GSE17563 data set

Finally, we downloaded the GSE17563 dataset through the GEO database, and the TIMER database was analyzed for the level of immune cell infiltration in each sample. We found that H19 expression was also negatively correlated with B-cell infiltration and positively correlated with macrophage infiltration in the GSE17563 dataset (Fig. 8A). Survival analysis showed that H19 and high macrophage expression also had poor OS (Fig. 8B). Although the B-cell infiltration level was not significantly different from the survival prognosis, a high B-cell invasion level had a better prognosis than a low–high infiltration level (Fig. 8B).

The above results indicate that high expression of H19 has a poor prognosis in patients with colon cancer, of which B-cell infiltration and macrophage infiltration may play an important role.

### Construction and analysis of the H19 regulatory network based on the TCGA database

**Fig. 9:**
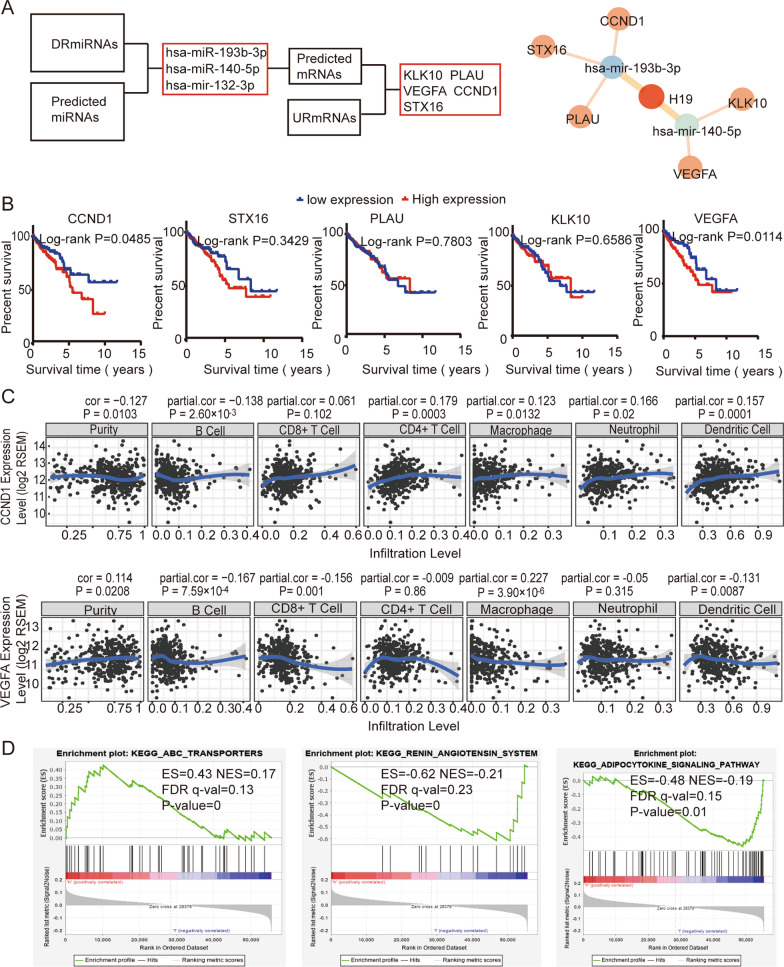
Construction of the H19 ceRNA network based on TCGA database. **A** Flow chart of constructing the H19-miRNA-mRNA network. Two H19-miRNA pairs and five miRNA-mRNA pairs were finally identified, and the network was visualized by Cytoscape software. **B** Prognostic analysis of five mRNAs in the ceRNA network. This result shows that the expression of CCND1 and VEGFA is significantly related to the survival prognosis of colon cancer. **C** Correlation analysis between mRNA (CCND1 and VEGFA) expression and six immune cell infiltration levels based on the TIMER database. **D** Gene set enrichment analysis for H19 expression. The results showed that patients with high H19 expression were mainly concentrated in ABC transporters. Patients with low H19 expression were mainly concentrated in the renin angiotensin system and adipocytokine signaling pathway. Downregulated miRNA (DRmiRNA), Upregulated mRNA (URmRNA)

Then, we constructed the H19 regulatory network based on the lncRNA competitive endogenous RNA mechanism (Fig. 9A). We first screened 150 miRNAs (Additional file 5: Table S5) whose expression was downregulated in colon cancer. According to the miRCode database, we predicted that H19 might target targeted miRNAs. Three miRNAs (miR-193b-3p, miR-140-5p and miR-132-3p) that could target H19 were obtained by taking the intersection of these two sets. Next, the miRDB, TargetScan and miRTarBase databases were used to predict the binding target mRNAs and then intersected with 1148 upregulated mRNAs (Additional file 6: Table S6). Finally, we obtained two lncRNA-miRNA pairs (H19-miR-193b-3p and miR-140-5p) and five miRNA-mRNA pairs (miR-193b-3p-CCND1, STX16, PLAU; miR-140-5p-KLK10, VEGFA). Data were visualized with Cytoscape 3.7.1 software.

Next, we analyzed the prognosis of five mRNAs in this network in patients with colon cancer (Fig. 9B). The results showed that the high expression of CCND1 and VEGFA was significantly correlated with poor prognosis of colon cancer (*p* < 0.05). High expression of CCND1 may lead to resistance to many antitumor drugs (Additional file 7: Figure S1). The high expression of CCND1 and VEGFA was positively correlated with the level of macrophage infiltration (*p* < 0.05) based on the TIMER database (Fig. 9C). This is consistent with the high expression of H19 (Figs. 7B, 8A). GSEA revealed that high expression of H19 was mainly concentrated in ABC transporters (Fig. 9D), and H19 low expression was mainly concentrated in the renin angiotensin system and adipocytokine signaling pathway (Fig. 9D).

### The expression of CCND1 and VEGFA were regulated by H19 and were significantly related to the infiltration of M2 macrophages

**Fig. 10:**
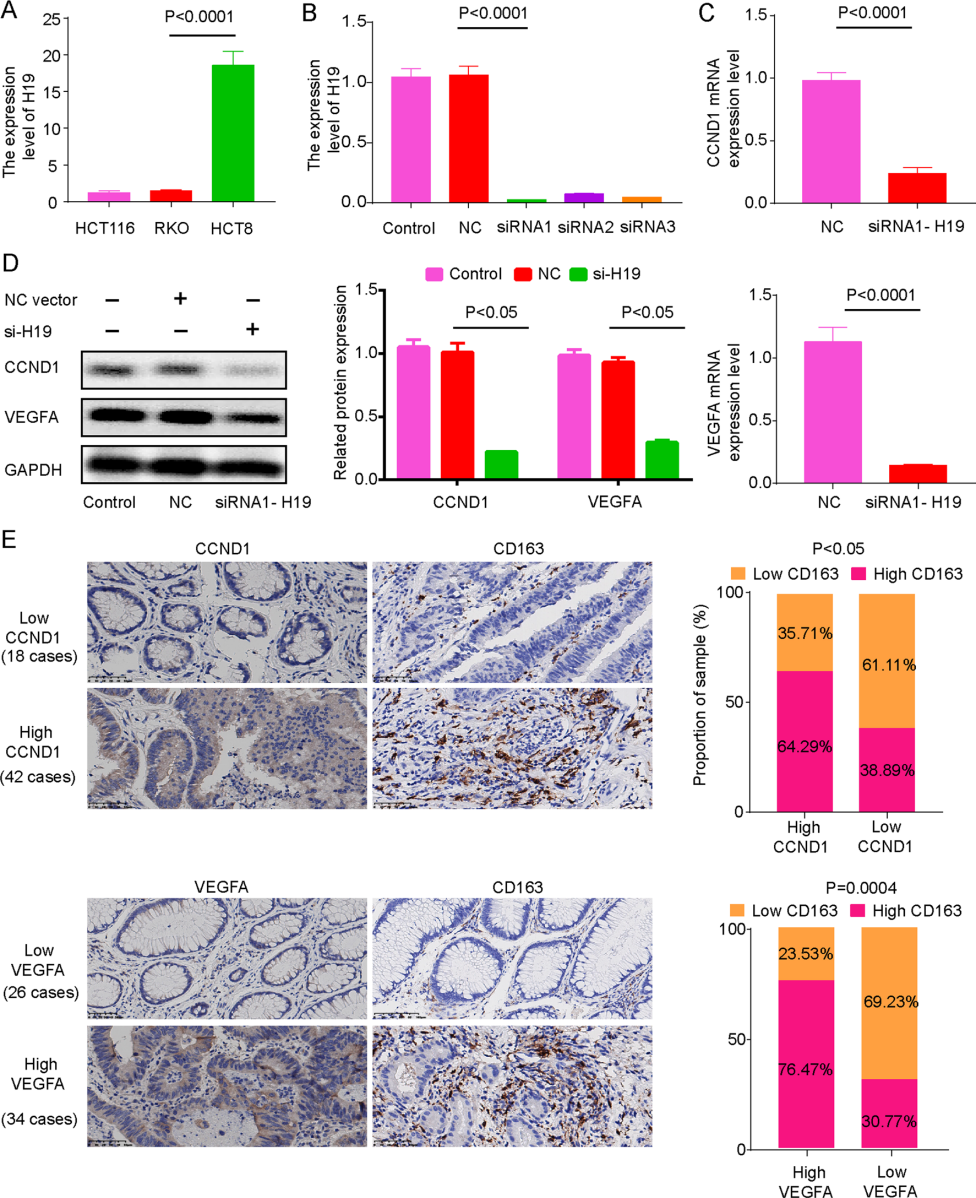
Knockdown the expression of H19 inhibits the expression of CCND1 and VEGFA. **A** H19 was significantly higher in HCT8 than HCT116 and RKO (Student's t-test). **B**, **C** Knockdown the expression of H19 significantly inhibits the expression of CCND1 and VEGFA mRNA (Student's t-test, P < 0.05). **D** Knockdown the expression of H19 significantly inhibits the expression of CCND1 and VEGFA protein (Student's t-test). **E** The expression of CCND1 and VEGFA protein were significantly correlated with M2 type macrophages (Chi-square test)

In order to further verify the above results, we screened out the HCT8 with the highest H19 expression in three colorectal cancer cell lines (Fig. 10A). After knocking down the expression of H19 in the HCT8 cell line, we found that the expression of CCND1 mRNA and VEGFA mRNA were significantly down-regulated (Fig. 10B, C). Western blot experiments further confirmed that knocking down the expression of H19 significantly inhibited the expression of CCND1 protein and VEGFA protein (Fig. 10D). Finally, further analysis of immunohistochemical experiments found that the level of M2 macrophage infiltration in colorectal cancer tissues with high expression of CCND1 or VEGFA was also significantly increased (Fig. 10E).

## Discussion

Colon cancer is a high-incidence gastrointestinal tumor [1], and its poor prognosis is related to the TME [36, 37]. Many previous studies have identified colon cancer subtypes based on genomic maps [38–40]. However, few studies have investigated colon cancer analysis based on immune characteristics. In this study, we identified three subtypes in colon cancer by using ssGSAE and cluster analysis (Fig. 1A). By analyzing the expression levels of HLA genes and immune checkpoint genes in the three subtypes, we found that the expression of HLA genes and PD-L1 gene in Immunity_H was significantly higher than that in the other two subtypes (Fig. 1B). This result suggests that patients with the Immunity_H subtype may be able to obtain greater benefits from the treatment of PD-L1 inhibitors.

A study has reported that interleukin 27 regulates lncRNA and mRNA expression in human macrophages [41]. Xu et al. reported that lncRNA Sros1 can activate innate immune response [42]. Previous studies have found that lncRNA ITPRIP-1 activates MDA5 to regulate the immune response [43]. These studies show that lncRNA expression can regulate changes in the IMand affect the disease process. We therefore further investigated the lncRNAs that are dysregulated in three colon cancer subtypes. The lncRNAs (Fig. 3A, B) that intersected with the immune-related lncRNAs were screened out, and we identified 35 lncRNAs related to immune infiltration (Fig. 3C). Cox regression analysis identified an immune infiltration-related 5-lncRNA signature (Fig. 3D, E). The results show that the signature can well evaluate the prognosis of colon cancer patients and is also an independent prognostic factor (Fig. 4A–C). Finally, we used this signature to calculate the risk score formula for each sample and combined the risk score with clinical factors (age, gender, and TNM stage) to obtain a combined model (Fig. 5B, C). This model can effectively improve the prediction of OS for colon cancer patients. GSEA results indicate that the main enrichment items of KEGG in high-risk patients are the Wnt signaling pathway and mTOR signaling pathway. The main enrichment items of KEGG in low-risk patients were the cytosolic DNA sensing pathway and Toll-like receptor signaling pathway (Fig. 6B). The Wnt signaling pathway and mTOR signaling pathway have been widely reported to be related to cancer progression [44–46], and these two pathways have been reported to be related to tumor immune escape and immune checkpoint inhibitor resistance [47]. Their activation promotes better survival and development of tumor cells. The Toll-like receptor signaling pathway is closely related to the regulation of the IM [48, 49], which is consistent with our findings.

Next, we analyzed each lncRNA and found that the expression of H19 was significantly related to the T stage, N stage, M stage and TNM stage of colon cancer (Fig. 6C), which suggested that high expression of H19 may be involved in the progression of colon cancer. Later, we found that H19 was significantly overexpressed in gastrointestinal tumors based on the TIMER database (Fig. 7A) and that H19 expression was related to B-cell and macrophage infiltration (Fig. 7B) and may be involved in the poor prognosis of colon cancer (Fig. 7C). The GSE17536 dataset further validates our results (Fig. 8). Related research reports indicate that H19 gene mutations are linked to the risk of colorectal cancer [50]. A previous study also reported that lncRNA H19 activates Wnt signaling and promotes epithelial-mesenchymal transition in colorectal cancer cells [51]. This evidence indicates that H19 is involved in the process of colon cancer and may be an important biomarker.

To explore the regulatory mechanism of H19 in colon cancer, we constructed H19-miRNA-mRNA based on the lncRNA competitive endogenous RNA mechanism (Fig. 9A). We analyzed the relationship between mRNA expression and OS in this network, and we found that high expression of CCND1 and VEGFA is an indicator of poor prognosis for colon cancer (Fig. 9B) and that the expression of CCND1 and VEGFA is associated with macrophage infiltration (Fig. 9C). Finally, PCR experiments, western blotting and immunohistochemistry further confirmed this result (Fig. 10). This result suggests that H19 may regulate immune cell infiltration by regulating the expression of CCND1 and VEGFA but may need to be tested in subsequent experiments. Finally, GSEA results showed that H19, which is highly expressed, is involved in activating ABC transporters. A study found that ABC transporters can cause multidrug resistance in tumors [52]. High expression of H19 leading to antitumor drug resistance has been observed in colon cancer [53, 54]. This suggests that H19 may be involved in tumor drug resistance mechanisms. More interestingly, we found that high expression of CCND1 may lead to resistance to multiple anti-colon cancer drugs based on the GSCALite database (Additional file 7: Fig. S1), which contains 5-fluorouracil, methotrexate, cetuximab and so on. These results suggest that the high expression of H19 is a poor prognosis indicator for colon cancer and that high expression of H19 may incur resistance to tumor chemotherapy drugs by regulating CCND1. However, the specific mechanism needs to be explored in basic experiments.

## Conclusion

This study identified a signature based on three subtypes of colon cancer that can greatly improve the predictive ability for colon cancer prognosis. Further analysis revealed that the expression of H19 may macrophage invasion and lead to poor prognosis of colon cancer, and high expression of H19 may lead to tumor cell resistance by regulating CDDN1. This study helps to inform clinical decisions and understand the role of H19 in colon cancer while providing potential biomarkers for targeted treatment of colon cancer.

## Supplementary Information

**Additional file 1: Table S1.** The 29 immune signatures represented by 29 different gene sets.

**Additional file 2: Table S2.** Differentially expressed lncRNAs between Immunity_L and Immunity_M.

**Additional file 3: Table S3.** Differentially expressed lncRNAs between Immunity_L and Immunity_H.

**Additional file 4: Table S4.** Expression profile of immune-related lncRNAs.

**Additional file 5: Table S5.** Downregulated miRNAs in colon cancer tissues.

**Additional file 6: Table S6.** Upregulated mRNAs in colon cancer tissues.

**Additional file 7: Fig. S1.** Correlation between CCND1 expression and antitumor drug resistance in colon cancer.

## Data Availability

We declared that the data and materials in this study are provided free of charge to scientists for non-commercial purposes.

## Abbreviations

LncRNAs: Long noncoding RNAs
IM: Immune microenvironment
ssGSEA: Single sample gene set enrichment analysis
OS: Overall survival
TNM: Tumor-node-metastasis
TME: The tumor microenvironment
IRGs: Immune-related genes
AIC: Akaike information criterion
ROC: Receiver operating characteristic
GSEA: Gene set enrichment analysis
GO: Gene ontology
KEGG: Kyoto Encyclopedia of Genes and Genomes
HR: Hazard ratios
TIMER: Tumor immune estimation resource
