# Supplementary figures and images for: Prognostic value and immune infiltration of novel signatures in colon cancer microenvironment
Source: Cancer Cell Int. 2021 Dec 18;21:679. doi: 10.1186/s12935-021-02342-8 (PMC8684099; doi:10.1186/s12935-021-02342-8)

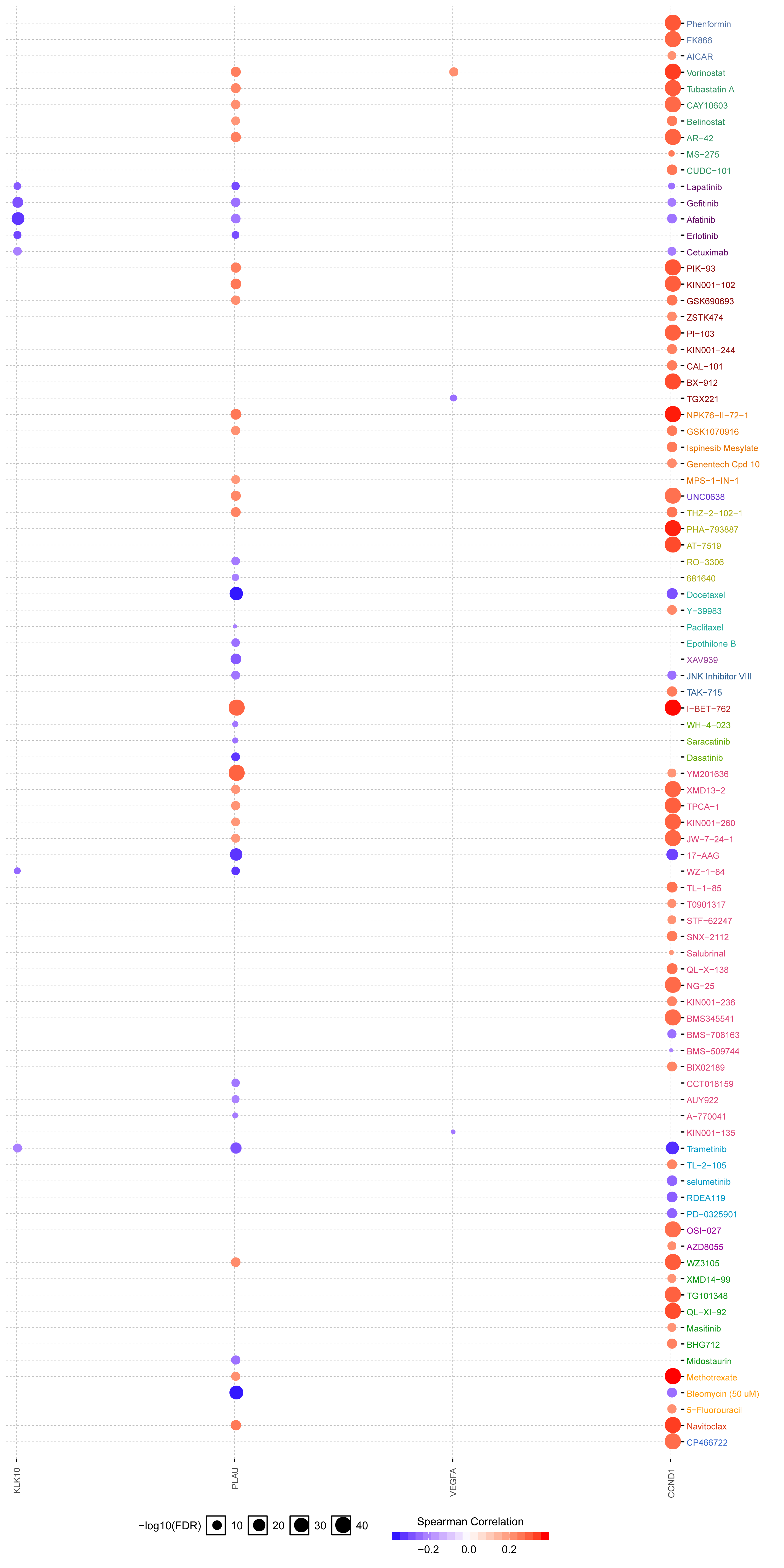

Supplement: Supplementary file 7 — Additional file 7: Fig. S1. Correlation between CCND1 expression and antitumor drug resistance in colon cancer. [file 12935_2021_2342_MOESM7_ESM.tif]
